# Structure and Catalytic Mechanism of Radical SAM Methylases

**DOI:** 10.3390/life12111732

**Published:** 2022-10-28

**Authors:** Tu-Quynh Nguyen, Yvain Nicolet

**Affiliations:** Metalloproteins Unit, Univ. Grenoble Alpes, CEA, CNRS, IBS, F-38000 Grenoble, France

**Keywords:** radical-based chemistry, methyl transfer, post-translational modification, iron–sulfur cluster, crystal structure

## Abstract

Methyl transfer is essential in myriad biological pathways found across all domains of life. Unlike conventional methyltransferases that catalyze this reaction through nucleophilic substitution, many members of the radical *S*-adenosyl-L-methionine (SAM) enzyme superfamily use radical-based chemistry to methylate unreactive carbon centers. These radical SAM methylases reductively cleave SAM to generate a highly reactive 5′-deoxyadenosyl radical, which initiates a broad range of transformations. Recently, crystal structures of several radical SAM methylases have been determined, shedding light on the unprecedented catalytic mechanisms used by these enzymes to overcome the substantial activation energy barrier of weakly nucleophilic substrates. Here, we review some of the discoveries on this topic over the last decade, focusing on enzymes for which three-dimensional structures are available to identify the key players in the mechanisms, highlighting the dual function of SAM as a methyl donor and a 5’-deoxyadenosyl radical or deprotonating base source. We also describe the role of the protein matrix in orchestrating the reaction through different strategies to catalyze such challenging methylations.

## 1. Introduction

Methyl transfer is a ubiquitous reaction that occurs in many biological processes across all kingdoms of life. While it may appear to be a minor change in a single macromolecule, methyl transfer can have significant effects on a living organism, including gene expression regulation, protein modification and the biosynthetic pathway of a variety of other metabolites and natural products [1,2,3,4]. The most common biological methyl donor is *S*-adenosyl-L-methionine (SAM) [5]. Conventional SAM-dependent methyltransferases catalyze methylation via an S_N_2 displacement mechanism [4,6]. This reaction typically involves a nucleophilic center, such as a nucleophilic carbon [7] or, more generally, a heteroatom such as oxygen, nitrogen or sulfur, attacking the methyl of SAM, which is electrophilic due to the electron-deficient nature of the sulfonium group [8,9,10]. Although weak nucleophiles such as sp^2^-hybridized carbon, in some instances, can also react via the S_N_2 mechanism [11,12], the reaction is sometimes much more challenging in terms of activation energy and therefore requires an alternative reaction mechanism. In 2011, Grove and coworkers established the dual use of SAM in the methylation of ribosomal RNA, where SAM is involved in both nucleophilic and radical chemistry [13]. Indeed, researchers since then have discovered an increasing number of enzymes that can overcome the poor nucleophilic feature of some sp^2^-hybridized carbons via radical-based chemistry, namely, radical SAM methylases (RSMases) [14]. 

**Figure 1:**
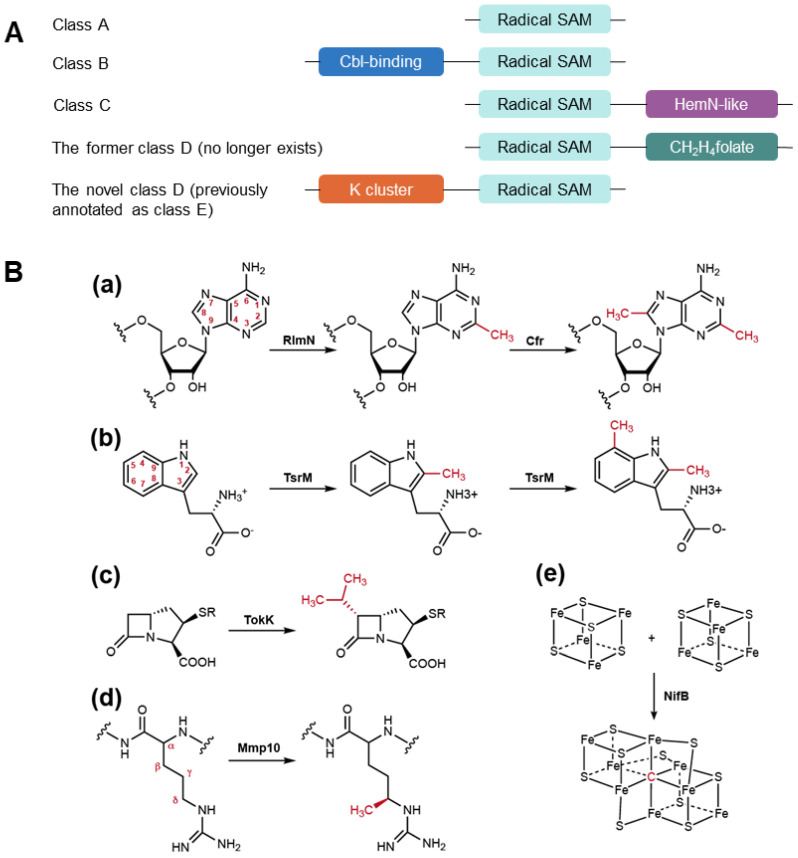
(**A**) The different radical SAM methylase (RSMase) classes containing different auxiliary domains; the radical SAM domain is omnipresent in all classes. (**B**) Selected reactions catalyzed by the RSMases discussed in this review, namely, RlmN and Cfr, TsrM, Tokk, Mmp10 and NifB.

RSMases are members of the radical SAM (RS) enzyme superfamily, the largest known enzyme superfamily, first described by Sofia and coworkers in 2001 [15,16]. With a conserved cysteine-rich motif, usually corresponding to CX_3_CX_2_C, with cysteine residues coordinating three iron atoms of a [4Fe-4S]^2+/+^ cluster, RS enzymes reductively cleave SAM to generate a 5′-deoxyadenosyl radical (5′-dA^•^) as a key catalytic intermediate [17,18,19]. In most cases, 5′-dA^•^ initiates radical-based reactions by abstracting a hydrogen atom (H^•^) from the substrate, leading to a remarkably wide range of transformations, including methyl transfers catalyzed by RSMases [20,21,22]. In a seminal review published in 2011, Zhang and coworkers classified RSMases into three different classes [23]. However, as new annotated sequences are continuously being discovered, novel RSMase classes have also been identified and, thus, have been discussed in different reviews [24,25,26]. Until very recently, there were five classes of RSMases described in the literature (classes A, B, C, D and E) based on their primary sequence, cofactor requisite and reaction mechanism (Figure 1A). Class A RSMases contain a single canonical radical SAM domain and use two conserved cysteine residues to catalyze methyl transfer to sp^2^-hybridized carbon centers [27,28,29,30]. Class B RSMases additionally carry an N-terminal cobalamin-binding domain to mediate methyl transfer between SAM and sp^2^- or sp^3^-hybridized carbons [23,31,32]. Class C RSMases contain a C-terminal domain with high sequence homology with the radical SAM enzyme coproporphyrinogen III oxidase HemN, which is able to catalyze methyl transfer to sp^2^-hybridized carbons using two SAM molecules simultaneously bound to the active site [23,25,33,34]. Until recently, it was believed that a class D RSMase exists that contains a methylenetetrahydrofolate-binding domain in addition to the radical SAM domain [25]. However, in 2022, in a very elegant work, Loyd and coworkers demonstrated that this protein was misannotated and instead corresponds to glycerol dibiphytanyl glycerol tetraether synthase [35]. Therefore, the former class D no longer exists. As a result, the last RSMase class, once designated as class E, has now become class D and comprises the NifB protein family, which is architecturally distinct from the other classes and catalyzes an unprecedented carbide insertion in the nitrogenase metallocofactor assembly [26].

Although we have learned a lot about RSMase-mediated methylation reactions in the last decade thanks to the enzymes’ crystal structure determination and to biochemical and spectroscopic studies, their mechanistic complexity still leaves numerous questions. The fact that RS-methylating enzymes are found in a plethora of biological pathways and that most members of the family still have not yet been characterized further emphasizes the importance of understanding their function, especially from a structural point of view. Given the large number of different RSMases existing in nature, this review by no means delivers an exhaustive scope of all enzymes in this family. Instead, we focus on the structure–function relationship of the RSMases and how the protein matrix can significantly influence the methylation strategy utilized by these enzymes. As a result, selected structurally characterized RSMases, including the most recent ones [27,31,32,36,37,38,39], are discussed in order to highlight their structural features and identify the key players in the different catalytic mechanisms.

## 2. RlmN and Cfr: How the Intriguing Chemistry of RSMases Was First Unveiled

**Figure 2:**
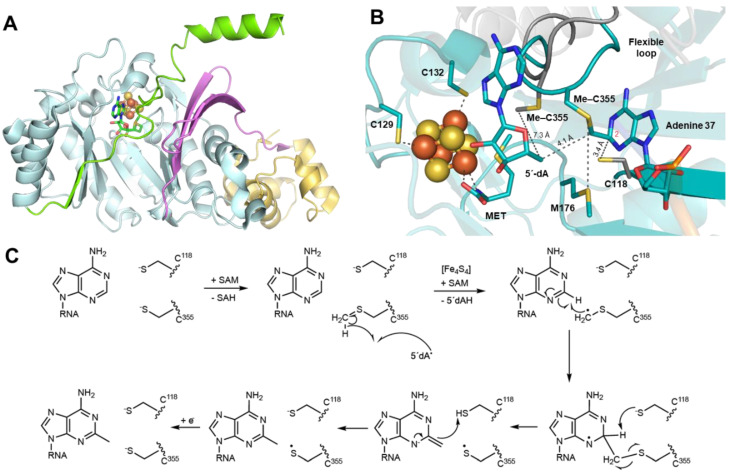
Structure of RlmN. (**A**) Overall RlmN structure composed of a radical SAM domain (light cyan), additional β strands (pink), an N-terminal helical domain (gold) and a C-terminal stretch (green). (**B**) Close-up view of the RlmN active site in the tRNA-crosslinked structure (PDB 5HR7) in the presence of 5′-dA and methionine (MET) (SAM cleavage products), depicted in teal. This structure is superimposed with the free RlmN structure (PDB 3RFA), depicted in gray. Please note that cysteine residue C118 shown in the figure was mutated into alanine in order to stabilize the crosslink between the protein C335 and the RNA substrate and was therefore not present in the actual structure [27,36]. (**C**) Proposed mechanism of C2 methylation on adenosine 2503 by RlmN.

RlmN and Cfr are two prominent members representing class A RSMases. The translational fidelity enhancer RlmN catalyzes not only the C2 methylation of the adenosine 2503 (A2503) position in the 23S subunit of ribosomal RNA [40,41] but also that of the adenosine 37 position of some transfer RNAs [42]. Cfr, on the other hand, despite sharing a high sequence similarity with RlmN, preferentially methylates the C8 position and, to a lesser extent, the C2 one of the same A2503 in 23S rRNA (Figure 1B), conferring resistance to multiple antibiotics [43,44,45]. Both of these methylated carbon centers are sp^2^-hybridized, making their homolytic C–H bond dissociation energies higher than that of a methyl group [46,47]. Therefore, a simple hydrogen abstraction from the substrate, as is typically the case for RS enzymes, might be energetically challenging. Instead, RlmN and Cfr transfer the methyl group to a conserved cysteine residue (C355 in RlmN and C338 in Cfr) of the protein before appending it to the substrate [28]. This methyl group is given by the first SAM molecule that binds to the active site, and then a second SAM arrives and undergoes reductive cleavage to generate the 5′-dA^•^ radical. The radical then abstracts hydrogen from methylcysteine to produce a protein-bound ^•^CH_2_-SCys intermediate [28]. From the classic methylation mechanistic reasoning, one might question the susceptibility of the cysteinyl residue to give up the methyl group due to its reduced electrophilicity compared to the positively charged sulfonium. However, it turns out that employing a nearby methyl carrier is a good strategy to overcome the substantial energy barrier in the activation of adenine sp^2^-hybridized carbons via radical chemistry [46]. Indeed, this route is thermodynamically advantageous in several respects. Firstly, the thiolate is a suitable nucleophilic carrier to acquire the methyl group of SAM via polar S_N_2 attack. Second, the bound sulfur atom further stabilizes the produced methylene radical intermediate. Third, the methylene radical addition to the poorly nucleophilic aromatic ring now becomes possible through the Minisci reaction mechanism [48]. Moreover, the X-ray structure of RlmN has shown that upon the binding of SAM to the active site, the cysteinyl sulfur atom is favorably located to be deprotonated by the nearby strictly conserved glutamate 105 residue (E105) and transform into the thiolate form, critical to initiating the reaction [27]. In this conformation, the methylated cysteine is also ideally located for methyl transfer from SAM [27] (Figure 2). 

RlmN and Cfr are salient examples of the dual use of SAM as a methyl donor and as a source of the 5′-dA^•^ radical but also of the use of a single SAM binding site for different functions, which represents an “economy in the evolution of binding sites” [13,27,28]. Indeed, instead of abstracting a hydrogen atom from the substrate sp^2^-hybridized carbon, RlmN and Cfr activate the methyl group to perform radical addition to the substrate double bond, allowing them to overcome the high activation energy barrier. Furthermore, both SAM molecules are recruited to the same binding site to participate in two distinct reactions. In addition, upon SAM binding, competition should occur between methyl transfer to C338 and SAM cleavage. The former may occur faster, and SAM cleavage would therefore only happen once C338 is methylated, allowing a single active site to play a dual role in supporting two distinct SAM cleavage modes [49] and highlighting the intimate relationship between the protein matrix and the order of reactions. Although the loop containing the methyl carrier cysteine residue is much less disordered in the SAM-bound structure than in the absence of SAM, the region remains flexible, and the methylcysteine located at the tip of the loop most likely confers the mobility required for the proposed “ping-pong” mechanism [27,28]. Accordingly, after picking up the methyl group and undergoing hydrogen abstraction by 5′-dA^•^, the methylene radical attached to the C355 residue attacks the sp^2^ C2 (or C8) of the adenosine ring, resulting in a covalently crosslinked protein–RNA intermediate. This covalent adduct has been observed in an X-ray structure of the RlmN-tRNA^Glu^ complex when the cysteine 118 residue is mutated to alanine [36] (Figure 2B). Indeed, mutagenesis studies have also shown that the conserved C118 residue in RlmN and the corresponding C105 in Cfr are indispensable in resolving the crosslink and are thus essential for the activity of these enzymes [29,50]. The observation of the covalent adduct in the crystal structure was consistent with previous EPR characterizations of both enzymes [30,47]. Subsequent isotopic labeling analysis in conjunction with density functional theory (DFT) showed that the covalent adduct contained an unpaired electron delocalized by the mesomeric effect throughout the adenine ring, with the spin density concentrated predominantly on adenosine nitrogen atoms N1 and N3 in RlmN and N7 in Cfr [30,47]. Interestingly, when interacting with tRNA, the methylated cysteine is brought closer to C5′ of 5′-dA^•^, that is, at a distance of 4.1 Å (instead of 6 Å in the wild-type RlmN structure), close enough for hydrogen abstraction by the 5′-dA^•^ radical [36], again showing the tight control of the surrounding protein matrix on the catalytic mechanism (Figure 2B). 

For several years after the first X-ray structure of RlmN was published in 2011, it was widely accepted that the formation of a disulfide bond between C188 and C355 resolves the crosslink between C355 of RlmN and C2 of the adenosine ring of the RNA substrate [23,24,27,51]. However, mechanistic studies by Silakov and coworkers in 2014 suggested that the resolving cysteine more likely acts as a general base to deprotonate C2 or C8 of A2503, although they did not rule out the potential formation of a disulfide radical anion species [30]. This finding was further supported by the abovementioned tRNA-bound RlmN structure, where the two key cysteines are located on opposite sides of the adenosine C2 position, with the mutated C118 pointing straight at this carbon [36] (Figure 2). The important role of deprotonation by C188 was also concluded from QM/MM calculations, demonstrating that this step is necessary to trigger the homolytic cleavage of the crosslinked intermediate through radical fragmentation [52]. The generated cysteinyl radical would then undergo a one-electron reduction for the next turnover by a currently unidentified source. Given the instability of this radical, C355 and C118 may form a transient thiosulfuranyl radical to prevent any side reactions, and the distance between this transitory radical and the [4Fe-4S] cluster could potentially allow for electron transfer between them [52]. Nevertheless, this suggestion remains hypothetical and requires further characterization to address. In the meantime, the cysteinyl radical would be stabilized by neighboring residues, particularly the conserved backbone methionine M176 [36]. Because the described interactions would mainly be selective for C2 methylation, and because Cfr and RlmN are considered distant relatives with non-interchangeable sequence regions [53], Cfr may employ a different strategy to structurally arrange the equivalent cysteines for C8 methylation. In fact, the strategy to employ a cysteine residue to finish the radical-based reaction has also been shown in spore photoproduct lyase (SPL), which is involved in DNA repair [54]. Indeed, a critical cysteine residue is necessary to provide a hydrogen atom and prevent side reactions of a highly reactive radical intermediate [55]. However, when this cysteine is mutated, SPL’s activity could still be restored by inserting another cysteine near the substrate in the active site [56]. This example demonstrates how the spatial geometry, not only of the active site but also of distant protein regions, can significantly influence the mechanisms that occur at the catalytic site.

## 3. Cobalamin-Dependent RSMases: Similar Domains, Different Mechanisms

**Figure 3:**
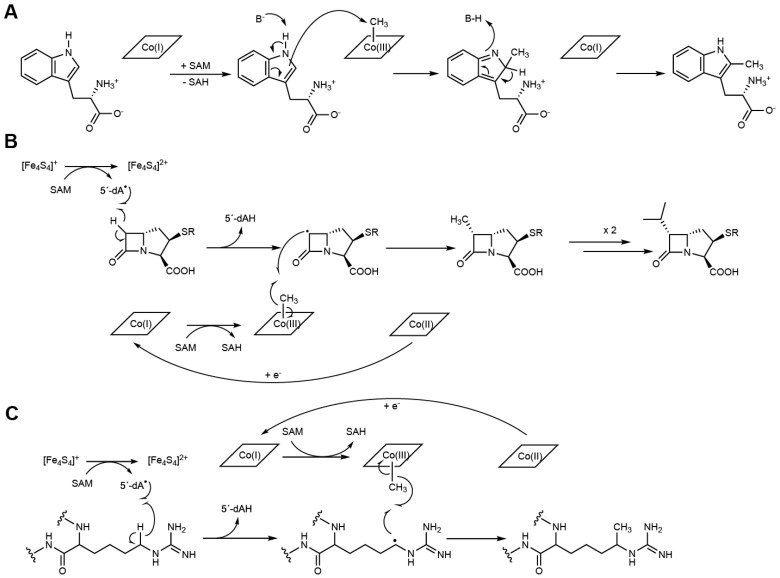
Proposed mechanisms for (**A**) TsrM, (**B**) Tokk and (**C**) Mmp10.

The N-terminal cobalamin-binding domain, alongside the radical SAM motif, is a structural hallmark of class B RSMases, although not all cobalamin (Cbl)-dependent radical SAM enzymes are methyltransferases [57]. Until 2020, very little was known about the mechanistic details of cobalamin-dependent RSMases, although results from the few systems that had been reported indicated that these enzymes most likely use multiple strategies to catalyze their reactions. It was not until 2021 that the first crystal structure of a cobalamin-dependent RSMase was determined. Indeed, it was the structure of TsrM [31], an L-tryptophan (Trp) methyltransferase that methylates the sp^2^-hybridized C2 carbon of the Trp indole ring, resulting in 2-methyltryptophan (2-MeTrp), a precursor to the quinaldic acid moiety of the thiopeptide antibiotic thiostrepton A [58,59]. Recently, isotopic labeling experiments combined with the use of tryptophan analogs and synthetic probe assays showed that TsrM is also able to carry out methyl transfer from SAM to the C4 of 2-MeTrp, producing 2,4-dimethyltryptophan [60,61], and to the N-propargylamine nitrogen atom of several tryptamine derivatives [61]. The first Cbl-dependent RSMase’s crystal structure was quickly followed by those of two other proteins in the family: Tokk and Mmp10 [32,39]. Tokk is involved in consecutive methylations to install an isopropyl group on the C6 carbon of the carbapenem antibiotic asparenomycin A [62,63], while Mmp10 catalyzes the post-translational methylation of the C_δ_ position of arginine residue R285 of methyl-coenzyme M reductase [64,65,66]. Even though TsrM, Tokk and Mmp10 all use the Cbl cofactor as an intermediate methyl carrier, they employ very different mechanisms to catalyze the methyl transfer reaction [67]. Strikingly, TsrM is an unprecedented exception to the radical SAM enzyme mechanistic paradigm that normally proceeds through 5′-dA^•^ radical generation [58,68,69]. Indeed, despite harboring a typical radical SAM domain, including the corresponding [4Fe-4S] cluster, TsrM catalyzes a non-radical-based reaction, which is thus far proposed to occur via two successive nucleophilic substitutions [70]. The first step involves a methyl transfer from SAM to cob(I)alamin (Co(I)-Cbl) to generate methylcob(III)alamin (MeCbl) and *S*-adenosyl-L-homocysteine (SAH). MeCbl is then attacked by C2 of Trp, assisted by the deprotonation of the N1 amine of the indole ring, to produce 2-MeTrp [69] (Figure 3A). In contrast to TsrM, Tokk and Mmp10 are proposed to use SAM in both nucleophilic and radical chemistry to catalyze methyl transfer. Accordingly, after the reductive cleavage of SAM, the resulting 5′-dA^•^ radical abstracts a hydrogen atom from the targeted carbon to generate a carbon-centered radical on the substrate. It is interesting to point out that, in contrast to TsrM, for which the targeted carbon is sp^2^-hybridized, both Tokk and Mmp10 target sp^3^-hybridized carbons. The corresponding C–H bond dissociation energies are significantly different (~350 kJ/mol for sp^3^-hybridized C_α_ to an amide, 373 kJ/mol for sp^3^-hybridized C_δ_ of arginine and 462 kJ/mol for sp^2^-hybridized C2 of Trp [71,72]), and only the sp^3^-hybridized carbon C–H bonds can be easily broken by the 5′-dA^•^ radical. The resulting C-centered radical then attacks the methyl group of MeCbl, inducing homolytic cleavage of the Co–C bond to yield the methylated product and Co(II)-Cbl. Subsequently, MeCbl is regenerated by methyl transfer from a second molecule of SAM after the reduction of Co(II)-Cbl to Co(I)-Cbl [32,39] (Figure 3B,C). In the case of Tokk, multiple successive methylation reactions are necessary to fully mount the isopropyl group [32].

**Figure 4:**
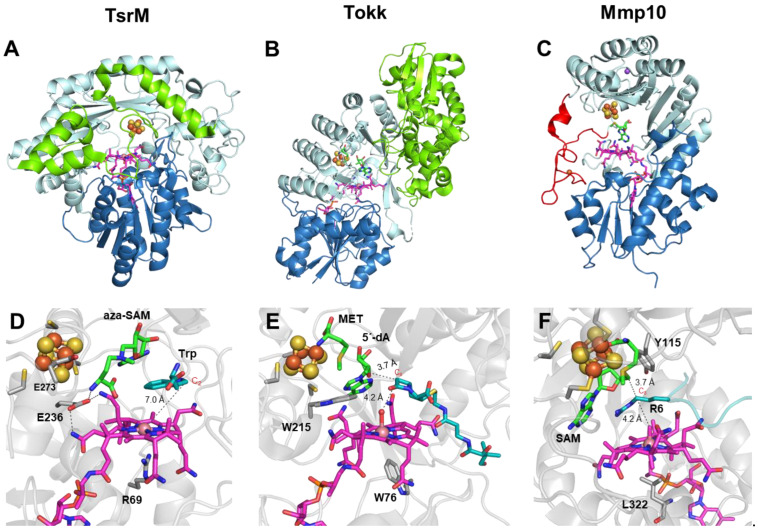
Structures of TsrM, Tokk and Mmp10. (**A**–**C**) TsrM, Tokk and Mmp10, respectively, shown as ribbon diagrams, colored by domain. The cobalamin-binding domain is shown in deep blue, with the cobalamin cofactor in magenta. The radical SAM domain is shown in light cyan, with the bound [4Fe-4S] cluster shown in orange and yellow. The C-terminal domains in TsrM and Tokk are shown in green. The iron loop in Mmp10 is shown in red. (TsrM: PDB 6WTE; Tokk: PDB 7KDX; Mmp10: PDB 7QBT). (**D**–**F**) Close-up view of the active sites of TsrM, Tokk and Mmp10, respectively. The cobalamin cofactor is depicted in magenta, and the bound [4Fe-4S] cluster is shown in orange and yellow. 5′-Azamethionine-5′-deoxyadenosine (aza-SAM), methionine (MET), 5′-deoxyadenosine (5′-dA) and SAM are labeled and shown in green. Substrates are colored in cyan (TsrM: PDB 6WTF; Tokk: PDB 7KDY; Mmp10: PDB 7QBS).

The non-radical catalytic nature of TsrM raised not only the question of “What defines a RS enzyme?” [68] but also the question of how the supernucleophilic Co(I)-Cbl is displaced by the weakly nucleophilic C2 carbon of Trp. The crystal structure of native TsrM, as well as that of TsrM bound to Trp and 5′-azamethionine-5′-deoxyadenosine (aza-SAM, an unreactive SAM analog), provided valuable insights into this mechanism (Figure 4A) [31]. Indeed, the structure revealed a conserved arginine 69 (R69) residue in the lower axial position of Cbl, likely playing an essential role in facilitating the polar displacement mechanism. It is worth noting that the nitrogen atoms of R69 are sufficiently far away from Co to not participate in its coordination but close enough to block water ligation, thereby keeping Cbl in an unstable pentacoordinated state [31] (Figure 4D). This observation is in line with previous EPR studies [68]. In addition, given the basic nature of the arginine side chain (pKa = 12.5), it is most likely positively charged. This, in turn, would help to stabilize the Co(I)-Cbl state while destabilizing MeCbl and thus favoring methyl acquisition by the weakly nucleophilic C2 carbanion. Indeed, a site-directed mutagenesis study showed that replacing R69 with lysine severely altered the enzyme’s activity, although methyl transfer from SAM to Co(I)-Cbl still took place, indicating that R69 specifically contributes to the Trp C2 methylation step [31]. 

Conversely, Cbl’s lower axial space in Tokk and Mmp10 is occupied by tryptophan (W76) and leucine (L322) residues, respectively [32,39] (Figure 4E,F). These two residues likely create a hydrophobic environment to prevent the stable hexacoordination of Co by water molecules or any other ligand. The W76 residue in Tokk exhibits a higher substitution tolerance compared to TsrM, with only a small loss of activity when mutated to phenylalanine, alanine or histidine, though its activity is reduced 50-fold with a lysine substitution [32]. Moreover, TsrM, Tokk and Mmp10 all appear to employ an amino acid residue to keep SAM and Cbl in juxtaposition and facilitate communication between them. In Tokk, the tryptophan residue W215 plays this role and is found at the analogous position with the glutamate residue E236 in TsrM; both are located on the radical SAM Cx_3_Cx_2_C-motif-containing loop, two residues downstream of the last cysteine residue [73]. In both cases, the residue sits in the median position and interacts with both the amino group of SAM and an acetamide side chain of Cbl (Figure 4D,E). Mmp10, on the other hand, uses the substrate arginine residue (R285 in methyl-coenzyme M reductase annotation) instead, coordinated in between SAM and Cbl, ideally located for a “pull-push” radical transfer mechanism, which has been supported by subsequent quantum mechanical calculations [74] (Figure 4F). Indeed, these proteins display structural flexibility to tightly control substrate binding and optimize the methyl transfer reaction. In Mmp10, the substrate residue R285 C_δ_ atom is sandwiched between the C5′ atom of SAM and the methyl group of MeCbl, which are ideal distances for both hydrogen abstraction by 5′-dA^•^ and subsequent methyl transfer from MeCbl [39]. Substrate binding induces an 11.6 Å displacement of the helix α1a and a 3.4 Å displacement of helices α1 to α4 of the radical SAM domain, leading to a closed conformation of the active site. In TsrM, a movement of the C-terminal loop by about 16 Å that closes off the Cbl-binding cavity also takes place upon substrate binding. However, in this conformation, the C2 position of the substrate Trp is not optimal for receiving the MeCbl methyl. This intermediate “flipped conformation” was explained by the use of OHCbl instead of MeCbl in the crystallization condition [31] and possibly by the fact that Trp should bind in different functionally relevant conformations in the active site to afford methylation at different indole ring positions [61]. In Tokk, the substrate-bound protein structure bears a strong resemblance to that of RlmN crosslinked with its substrate tRNA (see previous section). The relative positions between the targeted carbon atoms and the methyl carriers are indeed very similar. In RlmN, the methylcysteine residue is located at the same position as the hydroxyl group of OHCbl in the Tokk structure. Furthermore, their substrates occupy comparable positions in the active site [32,36]. These positions are appropriate for efficient hydrogen abstraction to activate methylcysteine and the carbapenem C6 position in RlmN and Tokk, respectively [32,36]. Moreover, the substrate-bound Tokk structure also supports the differences in rate constants established for each of the three methyl transfers carried out by this enzyme [62,75]. Indeed, the second methylation would be more favorable than the first, as the position of the new targeted carbon would be closer to the Cbl axial ligand. However, the presence of the ethyl carbon would then sterically hinder the addition of the third methyl group. This structural interpretation is consistent with the relative comparison of the three methylation rate constants *k*_3_ < *k*_1_ < *k*_2_ [32,62,75]. The substrate-binding channel of Tokk is distinct from that of TsrM and Mmp10 because there are no considerable conformational changes as observed in the latter two [32]. However, Tokk could also find its way to optimally position the β-lactam ring for hydrogen abstraction from C6 by 5′-dA^•^ and for subsequent methyl acquisition from MeCbl [32] (Figure 4E).

In addition to the Cbl- and substrate-binding domains, TsrM, Tokk and Mmp10 all accommodate a radical SAM core domain. A noteworthy feature of TsrM is that a glutamate residue (E273) coordinates the fourth iron atom of the radical SAM [4Fe-4S] cluster. This has been postulated to hamper SAM binding to the cluster and hence its homolytic cleavage to generate the 5′-dA^•^ radical [31]. However, the structures of other RSMases, such as Mmp10 and NifB (discussed later in this review), have also shown the complete ligation of the RS cluster, despite the radical nature of the reactions catalyzed by these enzymes [37,39]. Furthermore, when E273 in TsrM was mutated to alanine, the enzymatic production of 2-MeTrp was significantly reduced, but SAM could not be cleaved even in the presence of reductants such as sodium dithionite. These results confirmed the catalytic importance of E273 but also showed that it is not the key factor that would preclude TsrM from performing SAM cleavage [61]. This raises the question of why an RS folding domain is used to catalyze a non-radical reaction. Knox and coworkers hypothesized that the RS cluster would act as an electron conduit between the Cbl cofactor and external flavodoxin reductase, which has previously been shown to improve TsrM’s turnover rate [31,76]. Berteau and coworkers later demonstrated that by replacing the three cysteine residues of the RS motif with alanines, the Cbl cofactor switched from a base-off conformation, as demonstrated by XANES, EXAFS [61] and EPR [68], to a base-on conformation that could better preserve the Co(III)-Cbl state and thus disfavor Co-C bond dissociation [61]. These findings suggest that the RS domain is important for proper Cbl pentacoordination and further facilitates MeCbl heterolytic cleavage by TsrM. However, such a cysteine-to-alanine modification may broadly alter the overall structure, leading to this Cbl conformational change. Furthermore, with E273 preventing its binding to the [4Fe-4S] cluster, the carboxylate group of SAM is now closer to the substrates Trp and Cbl and is thus proposed to serve as the base to deprotonate N1 of Trp during the attack of C2 on MeCbl [31,69]. From an evolutionary point of view, it is possible that TsrM inherited from the past a structural combination that is efficient in localizing SAM and Clb close to each other, favoring methyl transfer. Because, in this particular case, the substrate itself becomes nucleophilic, the absolute requirement of radical-based chemistry may no longer be necessary.

**Figure 5:**
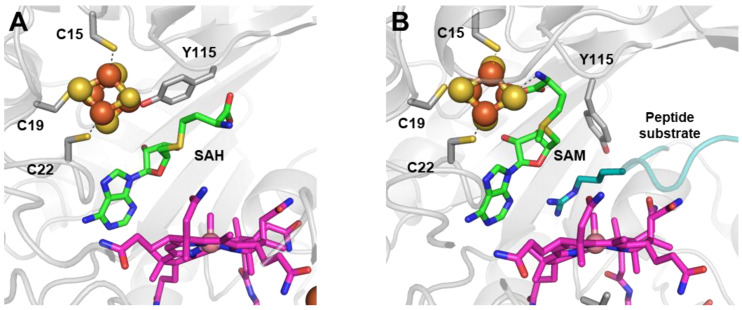
Mmp10 active site structure (**A**) with SAH in the absence of a substrate (PDB 7QBV) and (**B**) with SAM in the presence of a substrate (PDB 7QBS). Radical SAM domain residues are shown in light gray; the cobalamin cofactor is in magenta; the radical SAM [4Fe-4S] cluster is in orange and yellow; SAH and SAM are in green; and the peptide substrate is in cyan. Upon substrate binding, residue Y115 carried by the flexible loop does not bind to the unique iron, and the methionine moiety of SAM is hence able to bind to the cluster.

Mmp10 shares the fully ligated RS cluster feature with TsrM, although the fourth ligand is a strictly conserved tyrosine residue, Y115, causing SAM to be unable to bind to the unique iron [39]. However, in contrast to the E273 residue in TsrM, the Y115 residue does not prevent Mmp10 from performing radical chemistry. Indeed, upon substrate binding, likely aided by the reduction of the [4Fe-4S] cluster, Y115 dissociates from the cluster, allowing SAM to bind to the unique iron in the classic bidentate fashion. This suggestion is consistent with EPR experiments when adding SAM to reduced Mmp10 and the *S*-methyl-5′-thioadenosine (MTA)-bound Mmp10 structure [39]. For this reason, Y115, which is carried by a flexible loop, was proposed to act as a switch that allows the enzyme to distinguish between radical and nucleophilic chemistry according to the presence or absence of the substrate, without the need for two SAM-binding sites (Figure 5). This feature is reminiscent of that observed in NifB, where a flexible loop has been proposed to tune SAM cleavage depending on the presence of the substrate (see next section) [37,38]. Indeed, when SAM does not interact directly with the RS cluster, its methionine moiety displays a certain flexibility and can adopt different conformations to optimize the distance for a methyl transfer from SAM to Cbl. In the SAH-bound structure, the distance between the sulfur atom and cobalt is 3.3 A closer when compared to the structure of the MTA-bound Mmp10 structure [39]. The important role of the Y115 residue, notably its hydroxyl group in polar interactions upon substrate binding, was also demonstrated by the total loss of enzyme activity when replacing Y115 with alanine and the minimal activity detected when replacing it with phenylalanine [39]. In addition, Mmp10 contains a unique iron loop located under Cbl, which is proposed to play a role in the electron conduit from the Co. Notably, mutating the four cysteine residues of this loop to alanines abrogated substrate methylation activity, although a small amount of 5′-dA was still produced [39]. In contrast to TsrM and Mmp10, the RS cluster in Tokk binds to the methionine moiety in both the presence and absence of the substrate through its unique iron [32]. In the structures of Tokk in complex with methionine and 5′-dAH, also known as the products of SAM cleavage, the RS cluster and SAM-binding motifs (GE, ribose,“GXIXGXXE” and the β6 motif) appear conventional in function, as in most RS enzymes [73,77,78].

In summary, alongside the Cbl-binding domain that acts as the intermediate methyl carrier, the conservation of radical SAM folding in these three enzymes is probably proof of a common ancestor. However, this particular protein architecture has evolved to adopt different mechanisms and strategies required to catalyze different challenging reactions. In all cases, SAM seems to play at least two different roles in catalysis. For Tokk and Mmp10, as is the case for many other RSMases, SAM serves as a methyl donor and a 5′-dA^•^ radical source. In TsrM, SAM also functions in two roles, although in the second one, SAM is utilized in an untraditional way, acting as a catalytic base instead of generating the radical to abstract hydrogen.

## 4. NifB: A Small RSMase That Goes beyond Methyl Transfer 

NifB is a recently added member of the RSMase family and has been identified as the novel class D (see above) [79]. This relatively small protein, with less than 300 amino acids, catalyzes the exceptionally complex reaction of NifB cofactor (NifB-co or L-cluster) synthesis [80]. NifB-co is the key precursor to the catalytic cofactor FeMo-co of nitrogenase, an enzyme capable of reducing atmospheric nitrogen to ammonium and thus playing an important role in the global nitrogen cycle [81,82,83]. FeMo-co, characterized as a [7Fe-9S-C-Mo-*(R)*-homocitrate] center, is arguably one of the most sophisticated metalloinorganic cofactors in biology. The nature of the core carbide ion C^4−^, unprecedented in enzyme cofactors, remained unknown for many years, attracting significant attention from researchers across the field. It was not until 2011 that the identity of this central carbide was discovered using X-ray emission spectroscopy, high-resolution X-ray diffraction and electron spin echo envelope modulation studies [84,85]. This carbide ion was later shown to be derived from a methyl group of a SAM molecule [86]. Indeed, the introduction of the carbide ion is carried out by the radical SAM enzyme NifB, which concomitantly catalyzes the fusion of two [4Fe-4S] clusters and the addition of a sulfide ion to yield NifB-co, a [8Fe-9S-C] center [86]. For this reason, NifB is regarded as the key enzyme in the nitrogenase active site assembly machinery, known as the NIF (Nitrogen Fixation) machinery [87]. FeMo-co biosynthesis by the NIF machinery was described and discussed in a comprehensive review by Buren and coworkers [87]. Therefore, in this section, we limit the discussion to the structure–function relationship of the representative class D RSMase member NifB to fit the scope of this review. 

**Figure 6:**
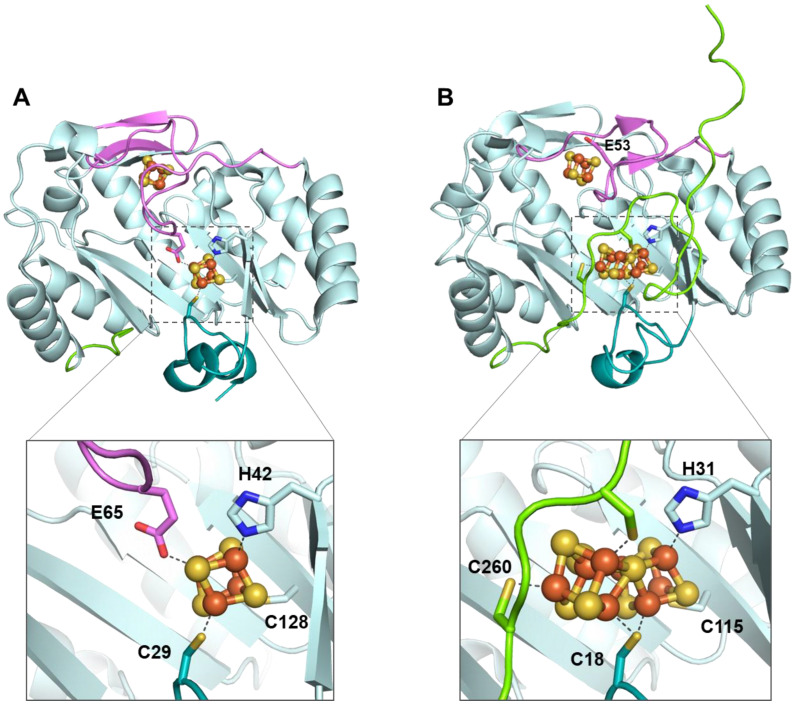
NifB structure accommodating (**A**) radical SAM and K1 clusters (PDB 6Y1X) and (**B**) radical SAM and [8Fe-8S] K cluster (PDB 7BI7). Residue E53 carried by the flexible loop in *Mth*NifB is also highlighted. The radical SAM domain is shown in light cyan, the N-terminal stretch is in teal, the C-terminal stretch is in green, the flexible loop is in pink, and iron–sulfur clusters are in orange and yellow.

**Figure 7:**
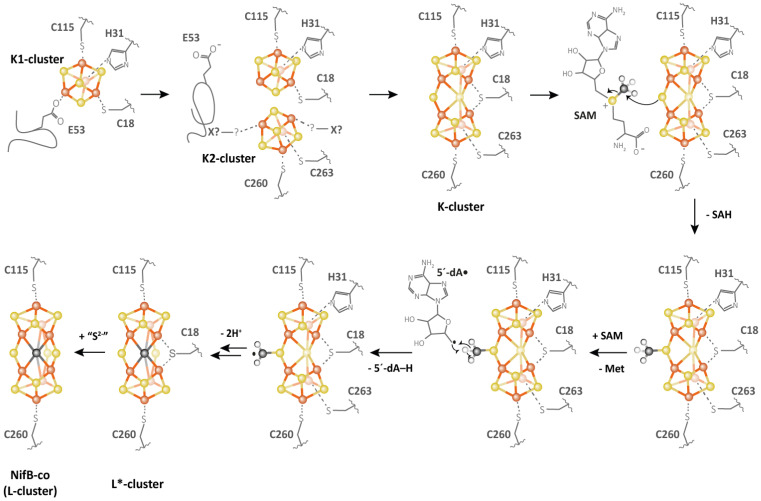
Proposed mechanism for NifB (adapted from Reference [90]).

NifB carries an N-terminal CX_3_CX_2_C radical SAM motif and also harbors two additional [4Fe-4S] clusters, termed K1 and K2, which serve as module substrates for the synthesis of NifB-co [88,89]. The first noteworthy feature of NifB is the fully ligated RS [4Fe-4S] cluster observed in the first crystal structure of NifB from *Methanotrix thermoacetophila* (*Mt*NifB) with only RS and K1 clusters (Figure 6) [37]. The labile K2 cluster was absent, resulting in important flexibility in the C-terminal stretch, which could not be observed in the electron density map. In this structure, the RS cluster is coordinated by the RS CX_3_CX_3_C-motif cysteine residues but also by a fourth strictly conserved residue, C62 (*Mt*NifB numbering), that binds to the unique iron [37]. Strikingly, C62 belongs to a short flexible loop that also contains the glutamate residue E65 ligating the K1 cluster. The latter is also bound to two cysteines (C29 and C128) and one histidine (H42) residue (*Mt*NifB numbering) (Figure 6A). Mutagenesis studies indicated that residues E65 and C62 present on the flexible loop both play a role in hampering SAM binding to the RS cluster and thus prevent futile SAM cleavage while NifB is still waiting for K2 cluster binding [37]. The fact that K1 and K2 clusters are co-substrates in NifB-co synthesis would explain not only their lability but also their susceptibility to further transformation once the protein’s redox state is properly tuned. Indeed, during the formation of NifB-co, residue H42 dissociates from the K1 cluster [89], allowing for ligand exchange and a certain degree of mobility of the K1 center required for cluster fusion. The K2 cluster, on the other hand, was proposed to be coordinated by two conserved cysteine residues, C273 and C276, which are located in the C-terminal stretch, which presumably becomes disordered when K2 is missing [38,88,89]. No further ligands have been identified so far for K2. This suggests that, unlike the RS and K1 clusters, which are sturdily attached to the protein matrix, K2 is more exposed to the surface of the protein [37], increasing its sensitivity to degradation. Nevertheless, EPR experiments have shown that NifB contains three distinct [4Fe-4S]^+^ clusters that are detectable upon reduction with dithionite [88,89]. In addition, the complete reduction of the fully loaded NifB is a prerequisite for SAM binding, methyl transfer and SAM cleavage reactions [89]. Furthermore, EPR experiments performed on NifB from *Methanosarcina acetivorans* (*Ma*NifB) in the absence of the K1 or K2 cluster suggested that one of the sulfide ions of the K2 cluster would receive the methyl via an S_N_2 nucleophilic mechanism, and the RS cluster is required for the homolytic cleavage of SAM [89,90]. Shortly after the first NifB structure was published, the structures of NifB from *Methanoacterium thermoautotrophicum* (*Mth*NifB) without metal clusters, as well as with RS and the putative K1 and K2 clusters, were reported [91] (Figure 6B). Consistent with the previous discussion, the cluster-deficient NifB form showed highly disordered regions containing ligands for the RS, K1 and K2 clusters, which are thus missing from the structure. The loaded NifB structure, on the other hand, appears to bind all three individual [4Fe-4S] clusters [91]. However, due to its modest 3.0 A resolution and inadequate refinement, the proposed structure was not satisfactorily convincing in terms of cluster coordination and geometry. A scrupulous reinvestigation of the crystallographic data led to a newly refined *Mth*NifB structure showing an already-fused [8Fe-8S] form that would correspond to the true K cluster (Figure 6B), reminiscent of the P cluster of the nitrogenase MoFe protein [38]. This K cluster is coordinated by a bridging cysteine residue, C18 (*Mth*NifB annotation), located on the N-terminal stretch, and two ligands for each symmetrical moiety, namely, C115 and H31, corresponding to K1 cluster ligands, and C260 and C263, corresponding to the proposed K2 cluster ligands [38]. Notably, C18 was initially a ligand to the K1 cluster only when K2 was missing [91]. In this K cluster structure, a bridging μ^2^-sulfide ion now points toward the RS cluster. This structural reinterpretation led to the conclusion that the [4Fe-4S] cluster fusion would occur first, and the K cluster would correspond to the substrate ready for methyl transfer and subsequent carbide insertion. Interestingly, when the K cluster is bound to the protein, the cysteine (C62) ligating the RS cluster’s unique iron is displaced, presumably allowing SAM to bind to the RS cluster to afford methyl transfer [38,91]. This observation further supported the hypothesis that the flexible loop, which provides ligands for both RS and K1 clusters, could act as a plug to control the binding and cleavage of SAM in the presence of the K cluster substrate [92]. This suggestion is very similar to the mechanism proposed for Mmp10, in which a tyrosine residue coordinating the unique iron would play a “switching” role to discriminate the nature of the reaction (methyl transfer or SAM cleavage) according to substrate binding (see the previous section). Collectively, these analyses have led to the following proposal of the NifB reaction mechanism: The K1 cluster would be accommodated by NifB via residues C115 and H31 located on the β strands of the RS domain, C18 on the N-terminal stretch and E53 on the flexible loop acting as an off switch to prevent SAM binding and cleavage, equivalent to the *Mt*NifB structure. Upon K2 cluster binding, the C-terminal stretch would bring residues C260 and C263 closer to the K2 cluster, thus providing ligation to the latter and putting the active site into a closed conformation. Next, K1 and K2 clusters would fuse together after the reduction of the clusters, previously proposed to be assisted by a ferredoxin (FdxN) [93], displacing the flexible loop from the cavity. SAM would then be able to bind to the active site and transfer a methyl group to the bridging μ^2^-sulfide ion of the K cluster, resulting in SAH dissociation from the RS cluster. The reductive cleavage of a second SAM molecule would yield the 5′-dA^•^ radical, abstracting a hydrogen from the K cluster-bound methyl. The subsequent deprotonation of the resulting methylene radical, potentially carried out by residue H42 acting as a base [94], and concomitant cluster rearrangement would generate the previously characterized [8Fe-8S-C] L* cluster [95,96]. Finally, a ninth sulfide would be added at the belt μ^2^-position occupied by C18. It has been shown that in vitro, this sulfide comes from the reduction of a sulfite ion, implicating an unknown mechanism [95,97] (Figure 7). However, this sulfite source remains highly improbable in vivo. Instead, a rhodanese-like protein often identified in the NIF regulon likely provides this sulfide as a cysteine persulfide [98].

This entire mechanism currently remains a subject of debate and awaits further experimental evidence to be elucidated. It is noteworthy that amongst the NifB sequences available, a few are limited to the minimal radical SAM domain, such as the ones discussed in this section. However, the majority additionally harbor a C-terminal NifX-like domain and, in fewer cases, an N-terminal NifN-like domain [99]. Even though the NifX-like domain was proposed to carry neo-synthesized NifB-co [100,101], it has been shown that this domain is not required for the proficient in vitro activity of NifB [99,102]. Indeed, once the prerequisite environment is established with a non-ligated unique iron, SAM is likely to bind in the classic bidentate fashion, as all SAM-binding motifs are present and appear to perform their traditional functions. However, this conventional SAM-binding mode would place SAM in an unfavorable position for methyl transfer to the bridging sulfide, as they would be on opposite sides of the K cluster [38]. In conjunction with the small size of NifB, this implies that SAM could potentially adopt different conformations within the same binding site to undergo different chemistry. This could lead to another outstanding example of the “economy in the evolution of binding sites” [27], where the protein not only uses a single site to bind two SAM molecules but also employs SAM to perform reactions far more complex than a single methyl transfer. However, the current lack of mechanistic understanding does not allow us to preclude an alternative methyl carrier, as in the case of RlmN and Cfr, instead of the nucleophilic bridging sulfide. Nonetheless, biochemical studies have supported a direct methyl transfer to the sulfur atom of the iron–sulfur cluster [86,90]. Because of the highly nucleophilic nature of the methylated substrate, as in TsrM (see previous section), NifB-catalyzed methyl transfer only requires an S_N_2 displacement mechanism rather than radical-based chemistry.

Overall, the biochemical, spectroscopic and structural characterizations of several NifB candidates have revealed important details about the mechanism of NifB-catalyzed reactions. Still, little is known about the full mechanism of cluster rearrangement and carbide insertion into the core of NifB-co. While evidence for a methyl transfer from SAM to a metal cluster sulfide ion allowed NifB to be classified as a new class of RSMases, the protein’s function is not limited to only methylation. Indeed, it is remarkable that NifB, the smallest enzyme discussed thus far, can perform such a complex radical-based mechanism to further generate the carbide ion and rearrange the cluster to form NifB-co. The detailed mechanisms of such events will strongly rely on future experimental research to be deciphered, particularly the determination of a high-resolution structure of K-cluster-bound NifB combined with spectroscopic data. 

## 5. Conclusions and Perspectives

In most cases, radical SAM methylases, despite their remarkable diversity of mechanisms and targeted substrates, share several key features to catalyze the same type of reaction. The first common feature is a methyl carrier that holds the methyl group. In RlmN and Cfr, a conserved cysteine residue is used as an intermediate methyl carrier; in cobalamin-dependent RSMases, the cobalamin cofactor plays this role; in class C RSMases, the enzymes use a second SAM and display two alternative SAM-binding sites; in NifB, the involvement of a transient methyl carrier remains to be studied. However, the methyl is first transferred to a sulfur atom of the K cluster before being activated by 5′-dA^•^. The second commonality is the homolytic cleavage of a second SAM to generate 5′-dA^•^. Except for TsrM, all known RSMases would likely use 5′-dA^•^ to abstract a hydrogen atom and activate either the SAM-derived methyl or the substrate. The protein matrix plays an essential role in fine-tuning the active site, hence selectively orienting the reaction to achieve a specific catalytic outcome. Lastly, these enzymes all appear to use SAM for at least two different functions: the first is as a methyl donor, while the second could be either as a source of the 5′-dA^•^ radical or as a general base involved in deprotonation. Because of its dual use, SAM is likely to bind in different modes in the same protein to undergo different transformations. For instance, RlmN uses a single SAM-binding site for both radical and nucleophilic chemistry. In Mmp10, although SAM binds to roughly the same location, the enzyme uses a more sophisticated mechanism to trigger each reaction in response to substrate binding, which is accompanied by structural changes in ligand coordination and domain folding. In NifB, the SAM-binding mode remains elusive despite efforts to incubate the protein with SAM prior to crystallization [37,91]. However, given the small size of NifB, the scenario of two distinct SAM-binding sites is highly improbable. However, this does not rule out the possibility of SAM binding in different conformations before and after methyl transfer. The structure of SAM-bound NifB would reveal important information about SAM’s binding mode. Regarding the SAM-binding site, it is also worth mentioning class C RSMases, which bind simultaneously to two SAM molecules, which are adjacent to each other in the active site [23,34]. Even though the mechanism of class C RSMases, specifically NosN, which is involved in nosiheptide biosynthesis, has been controversial in terms of radical intermediates and by-products [103], various studies have confirmed the direct interaction between derivatives of the two SAM molecules and thus the use of two distinct SAM-binding sites to catalyze methyl transfer [33,104,105].

How enzymes can catalyze a wide range of complex and thermodynamically unfavorable reactions that would not be feasible with chemically synthetic methods remains, to date, one of the most enthralling questions in biology. In this review, we have shown that a large part of the answer to this question may very well lie within the structure of the catalytic enzymes themselves through examples of RSMases catalyzing methyl transfers. Understanding the structure–function relationship of these enzymes could be extremely beneficial in the development of novel catalysts and drug discovery. For instance, the discovery of the strong medical implications of Cfr proteins may help overcome the antibiotic resistance threat. As another example, understanding the biosynthesis of the nitrogenase active site and the mechanism of the key component NifB may help inspire the development of more efficient catalysts for the production of ammonia, which, in turn, may have significant applications in the nutrition and energy-related fields [106]. More generally, the radical chemistry tightly controlled by the protein matrix could be harnessed to selectively functionalize inactivated C–H bonds and modify a variety of substrates [107]. The potential applications of RSMase-inspired chemistry are therefore promising but require extensive research for further characterizations of these enzymes.
